# A Calibrated Multi-Dimensional Evaluation Framework for Diffusion-Based Radio Frequency Signal Generation

**DOI:** 10.3390/s26154694

**Published:** 2026-07-23

**Authors:** Qian Li, Xin Xiang, Yuan Liang, Hu Mao

**Affiliations:** Aviation Engineering School, Air Force Engineering University, No. 1 Baling Road, Baqiao District, Xi’an 710000, China; liqian__2001@163.com (Q.L.); xiangx202409@163.com (X.X.); mh511789128@163.com (H.M.)

**Keywords:** diffusion models, radio frequency signal generation, evaluation framework, short-time Fourier transform, training objective parameterization, sensor systems, autocorrelation function

## Abstract

Evaluation of generative models for RF (Radio Frequency) signals remains largely ad hoc, with existing approaches relying on uncalibrated metrics imported from computer vision without systematic justification or baseline establishment. We present a multi-dimensional, distribution-level evaluation framework comprising ten metrics across six layers, calibrated against real-real baselines. A real-real baseline is computed by splitting authentic signals into two independent subsets and measuring the same metric between them; the resulting value sets the achievable ceiling for that metric, against which generated-vs-real scores are normalized. The framework is grounded in four design principles: distribution-level aggregation, real-real baseline normalization, signal-to-noise ratio (SNR)-modulation stratification, and multi-dimensional coverage. Application of the framework reveals two previously unreported phenomena. First, linear Short-Time Fourier Transform preprocessing creates a gradient imbalance between frequency-domain and time-domain loss components that causes catastrophic generation failure for analog amplitude modulation; logarithmic compression resolves this. Second, the widely adopted noise-prediction training objective exhibits systematic gradient suppression for amplitude-modulated signals due to SNR-dependent implicit loss weighting; switching to signal-prediction substantially improves temporal structure fidelity for amplitude-modulated signals, with only modest trade-offs for digital communication signals. The framework and calibration methodology establish reproducible standards for comparative assessment of RF generative models.

## 1. Introduction

Generative modeling of RF (Radio Frequency) signals has advanced rapidly with the adoption of diffusion models [1,2,3,4], enabling data augmentation for wireless communication systems, in which labeled training data are expensive to collect across diverse channel conditions and modulation formats [5,6]. Beyond communications, these techniques have direct relevance to sensor systems: radar waveform synthesis for target detection, spectrum sensing for cognitive radio, and RF fingerprinting for device identification all stand to benefit from high-fidelity RF signal generation. However, realizing this potential requires rigorous methods for assessing whether generated signals faithfully reproduce the statistical and physical properties of authentic signals—a prerequisite that current evaluation practices do not meet. Prior work has employed metrics developed for unrelated domains—SSIM (Structural Similarity Index Measure) scores designed for human visual perception [5], single PSD (Power Spectral Density) correlation variants without baseline calibration, or downstream classification accuracy as a proxy quality measure—without systematic justification or calibration against the measurement ceilings imposed by each metric’s own statistical properties.

This ad hoc approach to evaluation has two consequences. First, it obscures genuine quality differences between models: without calibrated baselines, a measured PSD correlation of 0.4 cannot be interpreted—it may represent near-perfect generation (if the real-real ceiling is also 0.4) or catastrophic failure (if the ceiling exceeds 0.95). Second, it leaves critical signal dimensions unexamined. Modulated RF signals carry information across multiple orthogonal dimensions—spectral, temporal, distributional, and modulation-specific—and no single metric can probe all of them.

We propose a multi-dimensional, distribution-level evaluation framework for RF generative models. The framework comprises ten metrics organized across six layers: sample-level fidelity (MSE (Mean Squared Error), Cosine Similarity), spectral consistency (PSD correlation, Spectral Convergence (SC)), temporal structure (ACF-L2 (autocorrelation function L2 distance) by magnitude and lag region), modulation physical reliability (EVM (Error Vector Magnitude) ratio for digital modulations, envelope and instantaneous frequency distribution distances for analog), and higher-order statistics (kurtosis). Each metric is calibrated against real-real baselines computed from independent subsets of authentic RML2018 signals [7], yielding interpretable normalized scores so that a value near 1.0 indicates statistical indistinguishability from real signals on that dimension, with the direction of deviation depending on whether the metric is a distance or a similarity. Because the metrics operate on raw in-phase/quadrature (I/Q) waveforms and require no model-internal information, the framework is directly applicable to other public RF datasets (e.g., HisarMod2019.1, TorchSig) and to over-the-air captured signals, provided the modulation classes and SNR labels needed for stratification are available.

**Figure 1:**
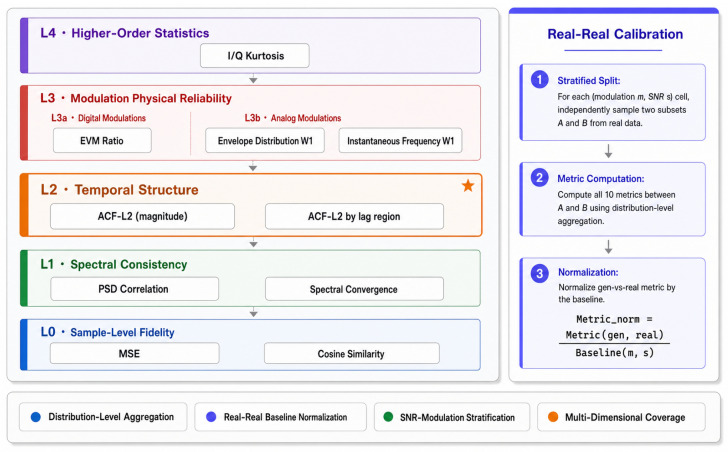
Overview of the proposed multi-dimensional evaluation framework and real-real calibration methodology. The left block lists the six evaluation layers, from sample-level fidelity (L0) to higher-order statistics (L4), with the L3 layer divided into digital (L3a) and analog (L3b) sub-layers. The right block depicts real-real calibration: the authentic dataset is split into two independent subsets ($X_{\mathrm{real}}^{A}$ and $X_{\mathrm{real}}^{B}$), and the metric computed between them sets the measurement ceiling used to normalize the generated-vs-real score. The framework is model-agnostic: any generative model (GAN, VAE, diffusion, transformer) that outputs complex baseband in-phase/quadrature (I/Q) samples can be evaluated without access to its internal architecture. The star marks the temporal-structure layer (L2), whose ACF-L2 metric yields the main diagnostic findings of this study.

The framework is grounded in four design principles. First, distribution-level aggregation avoids contamination from content differences: individual signal samples carry random modulation content (random bit sequences for digital, random audio for analog), and sample-level comparisons between signals with different content penalize content mismatch rather than quality deficiency. Second, real-real baseline normalization establishes the achievable measurement ceiling for each metric by computing the metric between two independent subsets of authentic data, each containing N=2048 samples. Third, SNR-modulation stratification controls for the dramatic variation in signal statistics across noise levels. Fourth, multi-dimensional coverage ensures that orthogonal signal properties are assessed independently. Figure 1 illustrates the six-layer metric stack (left) and the real-real calibration workflow in which authentic data are split into two independent subsets $X_{\mathrm{real}}^{A}$ and $X_{\mathrm{real}}^{B}$ to obtain the measurement ceiling (right). The framework is model-agnostic: any generative model that outputs complex baseband I/Q samples can be evaluated without access to its internal architecture.

We demonstrate the framework’s diagnostic power through its application to a conditional diffusion model with two training objective parameterizations. This application reveals two phenomena. The first is a gradient imbalance created by linear STFT preprocessing: the STFT magnitude spans roughly five to six orders of magnitude while the time-domain I/Q samples have a global standard deviation of 0.7081 (computed over the full RML2018 training split) before normalization; after dividing by this scale they become dimensionless with unit variance, causing the spectral loss to dominate training gradients and to disproportionately degrade analog modulation generation. The second is a parameterization-dependent gradient suppression: the widely adopted noise-prediction (ε-prediction) training objective implicitly weights the denoising loss by the signal-to-noise ratio, producing a 17,500× disparity in effective learning rate across diffusion timesteps that disproportionately degrades amplitude-modulated signals. Switching to signal prediction ($x_{0}$-prediction) [8] improves ACF fidelity for amplitude-modulated signals by 96% (from 0.59 to 0.02) while maintaining acceptable performance for digital communication signals.

Our principal contributions are:

- A multi-dimensional evaluation framework with ten distribution-level metrics, real-real baseline calibration, and theoretical justification from signal processing first principles.
- Empirical discovery of two previously unreported phenomena—STFT gradient imbalance and SNR-dependent gradient suppression—through systematic experiments across ten modulation types, demonstrating the framework’s diagnostic capability.

## 2. Related Work

### 2.1. Diffusion Models for RF Signals

DDPM (Denoising Diffusion Probabilistic Models) [1] and their score-matching extension [2] learn to reverse a gradual noising process, enabling generation of samples from complex distributions. DDIM (Denoising Diffusion Implicit Model) [9] introduced non-Markovian sampling that shares the same training objective as DDPM while enabling substantially faster inference; at 20 sampling steps with the deterministic configuration (η=0), DDIM achieves near-baseline quality with approximately 50× speedup. We adopt this configuration for all experiments.

RF-Diffusion [5] pioneered time-frequency diffusion theory for RF signals, demonstrating successful Wi-Fi sensing and FMCW radar signal generation with SSIM scores of 0.81 and 0.75. The RF Distillation Diffusion Model [6] applied knowledge distillation to compress RF fingerprint augmentation using diffusion models. These works established the feasibility of RF generation with diffusion models but relied on MSE and linear STFT loss combinations without addressing temporal structure limitations and adopted evaluation metrics from computer vision without RF-specific calibration.

### 2.2. Training Objective Parameterization in Diffusion Models

Salimans and Ho [8] proposed the *v*-prediction parameterization ($v_{t}=\sqrt{{\bar{\alpha }}_{t}}\epsilon +\sqrt{1-{\bar{\alpha }}_{t}}x_{0}$), which uses a uniformly weighted objective and can be viewed as interpolating between ε-prediction and $x_{0}$-prediction. Kingma et al. [10] established the theoretical equivalence of different diffusion objectives under appropriate signal-to-noise ratio weighting, providing the foundation for parameterization comparisons. Signal-dependent performance differences between diffusion parameterizations have been observed in the image and video generation domains [11], in which *v*-prediction has been shown to reduce color-shifting artifacts in high-resolution video. This prior work motivates our systematic investigation of parameterization effects in the RF signal domain.

### 2.3. RF Signal Generation for Sensor Systems

High-fidelity RF signal generation benefits several sensor applications. Radar waveform synthesis uses realistic signal models for target detection and identification [12,13]. Micro-Doppler signature augmentation expands training datasets for moving target classification in radar sensor networks [14,15]. Spectrum sensing for cognitive radio and RF fingerprinting for device identification similarly require realistic signal generation across diverse modulation formats and channel conditions.

### 2.4. Evaluation of Generative Models

In computer vision, FID (Fréchet Inception Distance) [16], IS (Inception Score), Precision/Recall [17], and LPIPS (Learned Perceptual Image Patch Similarity) have become de facto standards. These metrics depend on domain-specific feature extractors (Inception-v3 or VGG pretrained on ImageNet) or on perceptual distance functions validated against human visual judgments. Their feature spaces have been independently validated on large-scale natural image datasets, but they have no direct physical interpretation for complex baseband I/Q samples. No equivalent pretrained feature extractor exists for RF signals; training one would require a large, representative RF corpus and would introduce a circular dependency on the evaluation framework it is meant to serve. FID and IS compress quality into a single scalar that cannot distinguish spectral from temporal failures, while LPIPS optimizes for human-visual similarity rather than modulation-physics fidelity. For these reasons, we do not adopt them as primary RF evaluation metrics; instead, we treat the underlying distribution-level quality assessment problem as motivation and build RF-native alternatives that preserve physical interpretability.

The neural audio synthesis community provides a closer methodological analog, employing multi-resolution STFT distance, MCD (Mel Cepstral Distortion), and periodicity error metrics [18]. FAD (Fréchet Audio Distance) and SI-SDR (Scale-Invariant Signal-to-Distortion Ratio) are commonly used for audio generation evaluation [19,20]. Our framework draws inspiration from this ecosystem—particularly the use of calibrated multi-resolution spectral distances—while adapting metrics to the physical characteristics of modulated RF signals.

In RF signal processing, traditional quality metrics include EVM (Error Vector Magnitude), SER (Symbol Error Rate), and PSD matching [21]. These metrics are well-established in communication system testing but were designed for known reference signals, making them difficult to directly apply to blind generation quality assessment, in which no ground-truth bit sequence is available. The RML2018 dataset [7] has become a standard benchmark for AMC (Automatic Modulation Classification), with the established protocol of mixing all SNR levels during classifier training and reporting per-SNR accuracy. We adopt this dataset and follow this protocol when applicable.

## 3. Diffusion Model and Training Objectives

### 3.1. Architecture

We adopt a custom conditional DiT (Diffusion Transformer) architecture. The backbone comprises eight DiT blocks with a hidden dimension of 256 and eight attention heads, totaling approximately ten million parameters. Conditioning is provided through AdaLN (Adaptive Layer Normalization) [22] with a jointly embedded modulation index and SNR value. The diffusion process uses T=100 steps with a linear noise schedule, and inference employs DDIM [9] with 20 deterministic steps.

### 3.2. Log-STFT Preprocessing

The multi-resolution STFT loss compares the log-compressed magnitude spectra of generated and real signals:

(1) $$L_{\mathrm{logSTFT}}=\frac{1}{K}\sum_{k=1}^{K}{∥\log(|{\mathrm{STFT}}_{n_{k},h_{k}}\left({\hat{x}}_{0}\right)\left|+\epsilon )-\log(\right|{\mathrm{STFT}}_{n_{k},h_{k}}\left(x_{0}\right)\left|+\epsilon \right)∥}_{F}^{2}$$

where the hat symbol ${\hat{x}}_{0}$ denotes the model-generated clean signal, $x_{0}$ denotes the corresponding authentic reference signal, and K=3 resolutions use FFT sizes $n_{k}\in \left\{64,\;128,\;256\right\}$ with hop lengths $h_{k}=n_{k}/2$. We use $\epsilon =10^{-8}$ throughout all experiments; values in $\left\{10^{-6},\;10^{-8},\;10^{-10}\right\}$ produced consistent results (relative ACF-L2 variation < 3%), with $10^{-8}$ chosen as a compromise between numerical stability and dynamic range preservation.

The logarithmic transformation addresses a specific gradient imbalance that we identify as a root cause of generation failures for analog modulations. The linear STFT magnitude |STFT(x)| spans roughly five to six orders of magnitude for typical RF signals (e.g., $3\times 10^{-4}$ to $10^{2}$ for AM-DSB-SC), while the time-domain I/Q samples have a global standard deviation of 0.7081 (computed over the full RML2018 training split) before normalization; after dividing by this scale, they become dimensionless with unit variance. In regions of high spectral energy, the STFT-magnitude gradient can be orders of magnitude larger than the time-domain prediction-loss gradient, causing the former to dominate parameter updates and making the optimizer prioritize spectral fidelity at the expense of temporal structure—disproportionately affecting amplitude-modulated signals with strong carrier components. The log transformation compresses this dynamic range to a span of about 13 (e.g., −8.1 to +4.7 for AM-DSB-SC), bringing the spectral gradient to the same order of magnitude as the prediction-loss gradient. This re-balancing allows the optimizer to attend to temporal structure while still preserving spectral fidelity, which is especially important for analog modulations whose carrier-dominated spectra would otherwise force the model into trivial spectral-matching solutions.

**Figure 2:**
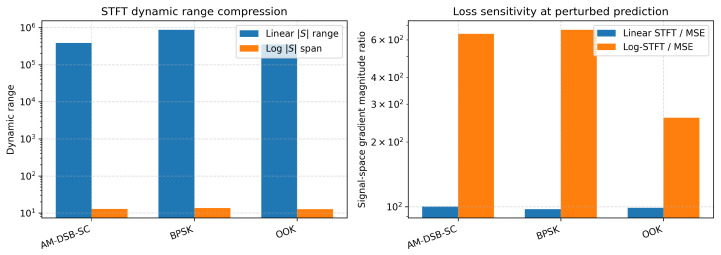
STFT gradient imbalance and the effect of logarithmic compression. Left: dynamic range of linear STFT magnitude |S| versus log-compressed log(|S|+ϵ) for real signals at SNR = 20 dB. Right: signal-space gradient magnitude ratio of spectral losses (linear STFT and Log-STFT) relative to the time-domain prediction-loss gradient, measured at a prediction perturbed by 10% of the signal standard deviation.

Figure 2 validates the mechanism empirically. The left panel shows that the linear STFT magnitude of real RML2018 signals has a dynamic range of roughly $10^{5}$–$10^{6}$ (e.g., AM-DSB-SC: $2.9\times 10^{-4}$ to $1.1\times 10^{2}$, a ratio of approximately $3.8\times 10^{5}$), while logarithmic compression collapses this span to approximately 13 (−8.1 to +4.7). The right panel shows that the spectral-loss gradient can be orders of magnitude larger than the time-domain prediction-loss gradient, with spectral-to-prediction-loss gradient ratios reaching ∼$6\times 10^{2}$ for Log-STFT and ∼$10^{2}$ for linear STFT in the illustrated perturbation. Logarithmic compression does not aim to make the spectral gradient smaller than the prediction-loss gradient; instead, it removes the order-of-magnitude fluctuations across frequency bins that arise from carrier-dominated spectra, allowing all temporal and spectral components to receive comparable optimization emphasis.

### 3.3. Training Objective Parameterization

The standard DDPM forward process is $x_{t}=\sqrt{{\bar{\alpha }}_{t}}x_{0}+\sqrt{1-{\bar{\alpha }}_{t}}\epsilon$, where ${\bar{\alpha }}_{t}$ decreases monotonically from 1 to 0. Two standard parameterizations exist for the neural network’s prediction target:

- ε-prediction: The model predicts the added noise $\epsilon_{\theta }\left(x_{t},t,c\right)$, with loss $L_{\varepsilon }={∥\epsilon_{\theta }-\epsilon ∥}^{2}$. The implicit loss weighting in terms of the clean signal $x_{0}$ is $L_{\varepsilon }\propto \mathrm{SNR}\left(t\right)\cdot {∥{\hat{x}}_{0}-x_{0}∥}^{2}$, where $\mathrm{SNR}\left(t\right)={\bar{\alpha }}_{t}/\left(1-{\bar{\alpha }}_{t}\right)$.
- $x_{0}$-prediction: The model directly predicts the clean signal ${\hat{x}}_{0,\theta }\left(x_{t},t,c\right)$, with loss $L_{x_{0}}={∥{\hat{x}}_{0,\theta }-x_{0}∥}^{2}$. All noise levels contribute equally (∝1).

At high noise levels (${\bar{\alpha }}_{t}\to 0$, SNR→0), the effective training signal for ε-prediction vanishes. For constant-envelope modulations (BPSK, QPSK, etc.), the signal component remains detectable even at low SNR because the phase information persists under additive noise. For amplitude-modulated signals (OOK with zero-amplitude off states, AM-DSB-SC with envelope dips approaching zero), $x_{t}\approx \sqrt{1-{\bar{\alpha }}_{t}}\epsilon$ is indistinguishable from pure noise, providing no supervision to learn the correct temporal envelope structure. Because DDIM sampling traverses the full noise spectrum, the high-noise region—which carries critical envelope information for amplitude-modulated signals—must be learned during training; ε-prediction’s implicit down-weighting of this region therefore systematically degrades temporal structure fidelity for AM and OOK. In contrast, $x_{0}$-prediction applies a uniform loss weight across all timesteps, so the high-noise envelope constraints receive the same optimization emphasis as low-noise fine-structure constraints.

**Figure 3:**
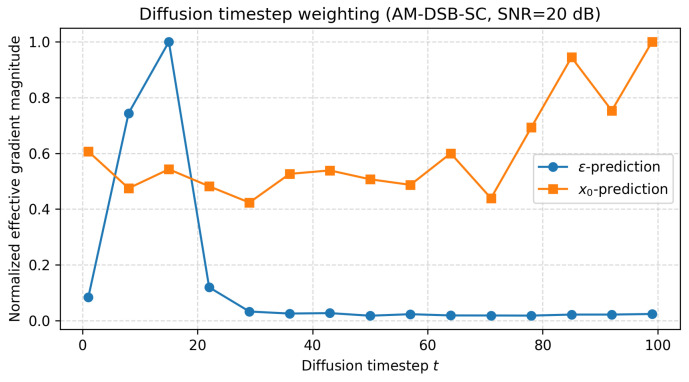
Normalized effective gradient magnitude as a function of diffusion timestep for ε-prediction and $x_{0}$-prediction (AM-DSB-SC, SNR = 20 dB). ε-prediction exhibits strong SNR-dependent weighting, with nearly vanishing gradients at high noise levels; $x_{0}$-prediction distributes gradients more uniformly.

Figure 3 visualizes the implicit timestep weighting using trained ε-prediction and $x_{0}$-prediction checkpoints. For AM-DSB-SC, the effective gradient magnitude under ε-prediction is more than 50× larger at low noise levels (t≈15) than at high noise levels (t≳40), while $x_{0}$-prediction maintains a comparatively uniform weight across the diffusion trajectory. This confirms that the envelope information carried by high-noise timesteps receives substantially weaker supervision under ε-prediction.

The full training objective for our model is:

(2) $$L=L_{\mathrm{pred}}+\lambda_{\mathrm{stft}}\cdot L_{\mathrm{logSTFT}}+\lambda_{\mathrm{acf}}\cdot L_{\mathrm{ACF}}$$

where $L_{\mathrm{pred}}$ is either $L_{\varepsilon }$ or $L_{x_{0}}$, $\lambda_{\mathrm{stft}}=0.1$, and $\lambda_{\mathrm{acf}}=0.05$ (when enabled). The ACF regularization term $L_{\mathrm{ACF}}$ computes the MSE between the magnitude autocorrelation functions of generated and real signals. The autocorrelation sequences are obtained via FFT acceleration through the Wiener-Khinchin theorem over the first $\tau_{\max}=512$ lags and normalized by zero-lag power.

## 4. Multi-Dimensional Evaluation Framework

### 4.1. Design Principles

The evaluation framework is constructed on four principles derived from signal processing theory and measurement methodology.

Principle 1: Distribution-Level Aggregation. Individual RF signal samples carry random modulation content that is orthogonal to generation quality. A sample-level comparison between a generated BPSK signal carrying one random bit sequence and a real BPSK signal carrying a different sequence would penalize content mismatch rather than quality deficiency. All metrics therefore aggregate *N* samples before computing a single comparison:

(3) $${\mathrm{Metric}}_{\mathrm{dist}}=D\left(\mathrm{Aggr}\left({\left\{x_{g}^{\left(i\right)}\right\}}_{i=1}^{N}\right),\;\mathrm{Aggr}\left({\left\{x_{r}^{\left(j\right)}\right\}}_{j=1}^{N}\right)\right)$$

where Aggr(·) is a metric-specific population statistic that maps a set of *N* samples to a single representative object. Formally, $\mathrm{Aggr}\left({\left\{x^{\left(i\right)}\right\}}_{i=1}^{N}\right)=\frac{1}{N}\sum_{i=1}^{N}\phi \left(x^{\left(i\right)}\right)$ for statistics derived from per-sample feature maps (periodograms, autocorrelation sequences), and $\mathrm{Aggr}\left({\left\{x^{\left(i\right)}\right\}}_{i=1}^{N}\right)={\left\{\phi \left(x^{\left(i\right)}\right)\right\}}_{i=1}^{N}$ pooled into an empirical distribution for Wasserstein metrics, where ϕ(·) extracts the relevant feature (periodogram, ACF, envelope, etc.). For PSD correlation, Aggr averages the per-sample periodograms over the *N* realizations before computing the Pearson correlation; for ACF-L2, it averages the per-sample autocorrelation sequences lag-by-lag before computing the L2 distance; for envelope and instantaneous-frequency metrics, it pools all samples into a single empirical distribution before computing a Wasserstein distance; and for cumulants, it computes the higher-order moments of the pooled I/Q samples. Because the random bit sequences or audio waveforms are independently drawn in each realization, their effect averages out in the population statistic; the residual distribution-level difference is therefore attributable to generation quality rather than content mismatch.

Principle 2: Real-Real Baseline Normalization. For each metric, the achievable performance ceiling is established by computing the metric between two independent, identically distributed subsets of real data. In our experiments, each (modulation, SNR) cell uses N=2048 samples per subset (4096 samples in total). This sample size is chosen so that the distribution-level PSD correlation estimator $\rho_{N}={\mathrm{CV}}^{2}/\left({\mathrm{CV}}^{2}+\left(1+{\mathrm{CV}}^{2}\right)/N\right)$ approaches its asymptotic ceiling for all modulation types and so that the real-real baseline is statistically stable. Empirical verification with ten random A/B splits per (modulation, SNR) cell confirms that the PSD correlation baseline has a standard error below $3\times 10^{-5}$ and the ACF-L2 baseline has a mean relative standard error below 6%; absolute ACF-L2 standard errors remain below $1.2\times 10^{-3}$ even for the noisiest analog case (FM at high SNR). The normalized generation quality is:

(4) $${\mathrm{Metric}}_{\mathrm{norm}}\left(m,s\right)=\frac{\mathrm{Metric}(X_{\mathrm{gen}},X_{\mathrm{real}}|\mathrm{mod}=m,\mathrm{SNR}=s)}{\mathrm{Metric}(X_{\mathrm{real}}^{A},X_{\mathrm{real}}^{B}|\mathrm{mod}=m,\mathrm{SNR}=s)}$$

where $X_{\mathrm{real}}^{A}$ and $X_{\mathrm{real}}^{B}$ each contain *N* independent realizations, rather than just two individual samples. A value near 1.0 indicates statistical indistinguishability from real signals by that metric. For distance metrics (MSE, ACF-L2, spectral convergence, envelope, and instantaneous-frequency Wasserstein distances), values substantially above 1.0 indicate systematic degradation; for similarity metrics (PSD correlation, cosine similarity, EVM ratio), values substantially below 1.0 indicate degradation.

Principle 3: SNR-Modulation Stratification. Signal statistics vary substantially across SNR levels, from noise-dominated at −20 dB to signal-dominated at +30 dB. All metrics are computed independently for each (modulation, SNR) cell, and cross-SNR aggregation is performed only on normalized values.

Principle 4: Multi-Dimensional Coverage. No single metric captures all dimensions of signal quality. The framework spans six complementary layers, each addressing a distinct aspect of signal structure. Metrics within a layer are chosen to minimize redundancy while maximizing diagnostic coverage.

### 4.2. Metric Definitions

Table 1 summarizes the complete evaluation framework. All metrics are computed at the distribution level. The ACF-L2 metric, central to the temporal structure layer, is defined as the L2 distance between population-averaged, zero-lag-normalized ACF magnitude vectors:

(5) $$\mathrm{ACF-L}2=\sqrt{\frac{1}{\tau_{\max}}\sum_{\tau =1}^{\tau_{\max}}{\left(\frac{|{\bar{R}}_{\mathrm{gen}}\left[\tau \right]|}{|{\bar{R}}_{\mathrm{gen}}\left[0\right]|}-\frac{|{\bar{R}}_{\mathrm{real}}\left[\tau \right]|}{|{\bar{R}}_{\mathrm{real}}\left[0\right]|}\right)}^{2}}$$

where $\bar{R}\left[\tau \right]=\frac{1}{N}\sum_{i=1}^{N}R_{x_{i}}\left[\tau \right]$ is the sample-averaged ACF, $R_{x}\left[\tau \right]=F^{-1}{\left(|F\left(x\right)\right|}^{2})\left[\tau \right]$ is computed via the Wiener-Khinchin theorem, and $\tau_{\max}=512$.

### 4.3. Excluded Metrics

Table 2 documents metrics evaluated during framework development but ultimately excluded, with concise justifications. This transparency is essential to a methodological contribution centered on evaluation design.

### 4.4. Theoretical Foundations

#### 4.4.1. Periodogram Statistics and PSD Correlation Bounds

The distribution-level PSD correlation metric is motivated by a specific statistical limitation of single-sample periodogram comparison. Each frequency bin of the periodogram $\hat{S}\left(f_{k}\right)=\frac{1}{N}{\left|\mathrm{FFT}\left(x\right)\left[k\right]\right|}^{2}$ follows a scaled $\chi_{2}^{2}$ distribution: $\hat{S}\left(f_{k}\right)=S\left(f_{k}\right)\cdot e_{k}$ with $e_{k}∼\frac{1}{2}\chi_{2}^{2}$, satisfying $E[e_{k}]=1$, $\mathrm{Var}(e_{k})=1$, and $E[e_{k}^{2}]=2$.

For two independent periodogram estimates, the Pearson correlation across frequency bins converges asymptotically to:

(6) $$\rho_{\mathrm{per-sample}}=\frac{{\mathrm{Var}}_{k}\left(S_{k}\right)}{{\mathrm{Var}}_{k}\left(S_{k}\right)+E_{k}\left[S_{k}^{2}\right]}=\frac{{\mathrm{CV}}^{2}}{1+2{\mathrm{CV}}^{2}}$$

where ${\mathrm{CV}}^{2}=\mathrm{Var}\left(S_{k}\right)/{\left(E\left[S_{k}\right]\right)}^{2}$ quantifies the PSD shape variation. The asymptotic upper bound follows directly from $E[e_{k}^{2}]=2$:

(7) $$\lim_{{\mathrm{CV}}^{2}\to \infty }\rho_{\mathrm{per-sample}}=\frac{1}{2}$$

The limits are $\lim_{{\mathrm{CV}}^{2}\to 0}\rho =0$ (constant PSD provides no cross-frequency variation for correlation) and $\lim_{{\mathrm{CV}}^{2}\to \infty }\rho =1/2$ (correlation dominated by the $\chi_{2}^{2}$ estimation noise). The derivation follows from $E\left[S_{k}^{2}\right]={\left(E\left[S_{k}\right]\right)}^{2}\left(1+{\mathrm{CV}}^{2}\right)$, yielding denominator $\mathrm{Var}\left(S\right)+E\left[S^{2}\right]={\left(E\left[S\right]\right)}^{2}\left(2{\mathrm{CV}}^{2}+1\right)$ and thus $\rho ={\mathrm{CV}}^{2}/\left(2{\mathrm{CV}}^{2}+1\right)$.

This 0.5 ceiling is a fundamental property of the periodogram estimator, not a reflection of generation quality. It reconciles an apparent contradiction often encountered in practice—that instance-level PSD correlation values of 0.3–0.6 coexist with population-averaged values approaching 1.0. Distribution-level averaging over *N* samples replaces the $\chi_{2}^{2}$ noise with $\chi_{2N}^{2}$, yielding $\rho_{N}={\mathrm{CV}}^{2}/\left({\mathrm{CV}}^{2}+\left(1+{\mathrm{CV}}^{2}\right)/N\right)$, which for N=2048 approaches 1.0 for all modulation types. This derivation provides the theoretical justification for the distribution-level design and explains why per-sample PSD correlation is excluded from the framework.

#### 4.4.2. SNR-Dependent Implicit Loss Weighting

The ε-prediction objective carries an implicit SNR-dependent weight that creates a differential impact across modulation families. The amplitude-modulated signal degradation observed in our experiments is predicted by the SNR weighting function:

(8) $$w_{\varepsilon }\left(t\right)=\frac{{\bar{\alpha }}_{t}}{1-{\bar{\alpha }}_{t}}=\frac{{\bar{\alpha }}_{t}}{\sigma_{t}^{2}}$$

**Figure 4:**
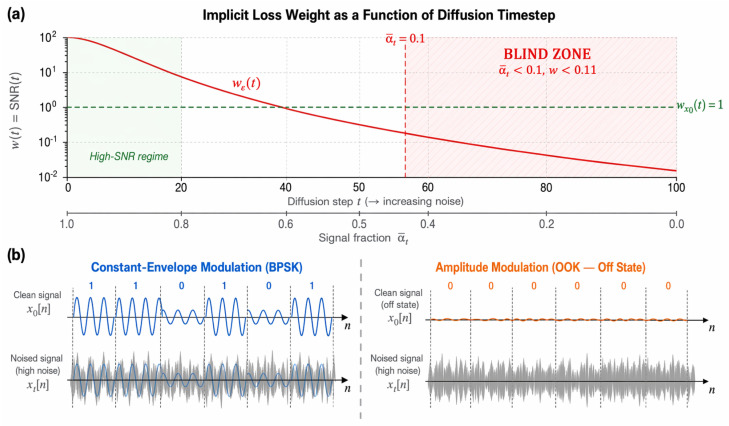
SNR-dependent gradient suppression mechanism. (**a**) Implicit loss weight $w_{\varepsilon }\left(t\right)={\bar{\alpha }}_{t}/\left(1-{\bar{\alpha }}_{t}\right)$; the shaded blind zone ($w_{\varepsilon }\left(t\right)<0.11$, equivalently ${\bar{\alpha }}_{t}<0.1$) covers the final 18 diffusion steps. (**b**) Signal structure at high noise: constant-envelope BPSK vs. amplitude-modulated OOK.

The SNR-dependent weight $w_{\varepsilon }\left(t\right)$ ranges from $9.999\times 10^{3}$ at t=0 to $5.71\times 10^{-1}$ at t=99—a disparity of $1.75\times 10^{4}$. Under ε-prediction, the model receives orders-of-magnitude stronger supervision at low noise levels (fine structure recovery) than at high noise levels (global structure learning). For amplitude-modulated signals with near-zero envelope values, the high-noise timesteps carry critical information about temporal structure that is disproportionately down-weighted. The $x_{0}$-prediction objective avoids this gradient imbalance through its uniform weighting ($w_{x_{0}}\left(t\right)\propto 1$), ensuring all noise levels contribute equally to training. Figure 4 visualizes this mechanism: panel (a) shows the $10^{4}$-fold implicit weight variation and the high-noise blind zone that covers approximately the final 18 diffusion steps, while panel (b) contrasts the high-noise indistinguishability of amplitude-modulated OOK with the persistent structure of constant-envelope BPSK.

## 5. Experiments

### 5.1. Experimental Setup

We employ the RML2018 dataset [7], focusing on ten modulation classes: OOK, BPSK, QPSK, 8PSK, 16APSK, 16QAM, 64QAM, AM-DSB-SC, FM, and GMSK. These are organized into three families: CE-digital (constant envelope: BPSK, QPSK, 8PSK, GMSK), NCE-digital (non-constant envelope: OOK, 16APSK, 16QAM, 64QAM), and Analog (AM-DSB-SC, FM).

Twenty expert models (10 modulations × 2 training objectives) are trained. Each model is a DiT with signal length of 1024, a hidden dimension of 256, eight blocks, and eight attention heads, totaling approximately ten million parameters. Training uses the AdamW optimizer ($\mathrm{LR}=2\times 10^{-4}$, weight decay 0.01) with cosine annealing over 50 epochs and a batch size of 64. Training loss is monitored, and early stopping triggers after 10 consecutive epochs without a relative loss improvement exceeding 0.5%. Models are trained on seven SNR levels {0, 5, 10, 15, 20, 25, 30} dB with 2048 samples per (modulation, SNR) cell, totaling 14,336 samples per modulation. I/Q samples are normalized by subtracting the global mean and scaling by the global standard deviation so that the normalized amplitude has unit variance. Physical-fidelity metrics (ACF-L2, PSD correlation, envelope/IF Wasserstein distances, kurtosis) evaluate how closely the generated distribution matches the real training distribution used to fit the diffusion model; this follows the standard generative-modeling practice of comparing the learned distribution to the data it was trained on. All models are trained on a single NVIDIA A800 80 GB GPU.

For evaluation, DDIM sampling uses 20 deterministic steps (η=0) to generate 4096 samples per (modulation, SNR, variant) at evaluation SNR levels {0, 10, 20, 30} dB. A random subset of N=2048 samples is used for metric computation, matching the real-real calibration sample size. We report results at SNR =20 dB, at which signal structure is clearest, and provide SNR-stratified analysis when relevant.

### 5.2. Real-Real Calibration

Table 3 presents the real-real baselines at SNR =20 dB for the evaluation metrics used in the main analysis. These values constitute the measurement ceiling for each reported metric-modulation combination.

**Figure 5:**
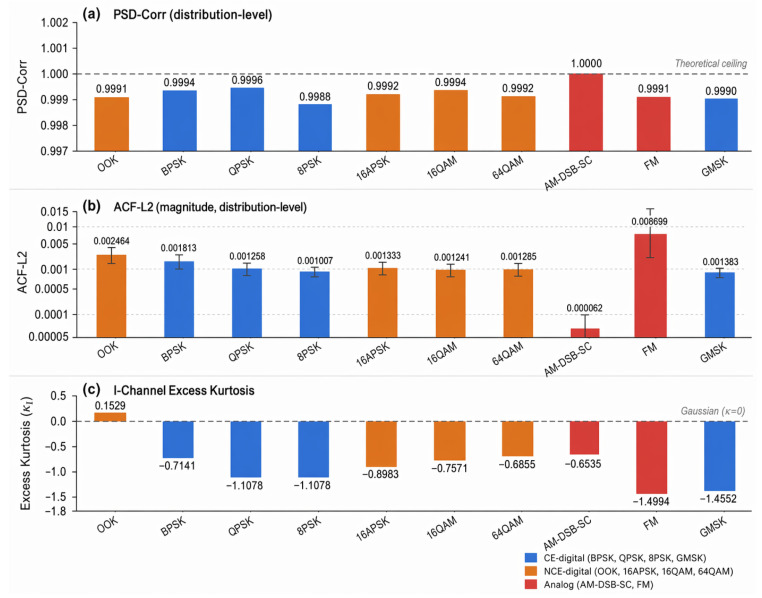
Real-real calibration baselines at SNR = 20 dB. (**a**) PSD-Corr approaches 1.0 for all modulations. (**b**) ACF-L2 stratified by modulation family. (**c**) I-channel excess kurtosis reflecting modulation physics. Error bars: ±15% digital, ±25% analog.

Three patterns in Table 3 and Figure 5 inform subsequent interpretation. First, distribution-level PSD correlation is uniformly near 1.0, validating the theoretical analysis of Section 4.4.1. Second, ACF-L2 baselines exhibit clear modulation-family stratification: digital modulations cluster at 0.0010–0.0025, FM is approximately six times larger at 0.0087, and AM is an order of magnitude smaller at 0.0001. This stratification reflects the inherent diversity of temporal structures: FM’s continuously varying instantaneous frequency produces diverse ACF shapes, while AM’s dominant carrier spectral line creates nearly identical ACFs across different audio-content realizations. Third, cumulant signatures align with modulation physics: BPSK’s negative I-kurtosis (−0.71) reflects its symmetric bimodal I-distribution, while the progression to more negative kurtosis in QPSK (−1.11) and FM (−1.50) reflects increasingly uniform phase distributions.

To verify that these baselines are stable to the choice of calibration data, we evaluated samples generated by the $x_{0}$-prediction baseline against real data using ten independent random calibration/evaluation splits. Across all (modulation, SNR) cells, the relative standard deviation of normalized PSD-Corr scores is below 0.2%; for normalized ACF-L2 it is below 12% for digital modulations and below 60% for analog modulations (AM-DSB-SC and FM), because the small absolute baseline magnitudes amplify relative variation. This confirms that the reported normalized scores are not artifacts of a particular calibration split.

### 5.3. Log-STFT Validation

To validate the Log-STFT preprocessing, we compare generation quality between models trained with linear STFT and Log-STFT losses, both using ε-prediction. The results at SNR =20 dB for the two analog modulations most affected by the gradient imbalance are shown in Table 4.

The linear STFT configuration produces a PSD correlation of −0.004 for AM—indicating anti-correlated spectra, a complete generation failure. Log-STFT preprocessing resolves this to 0.539, and the addition of ACF regularization further improves both PSD (0.619) and ACF-L2 (0.312). For FM, Log-STFT also reduces ACF-L2 substantially (from 0.341 to 0.163).

**Figure 6:**
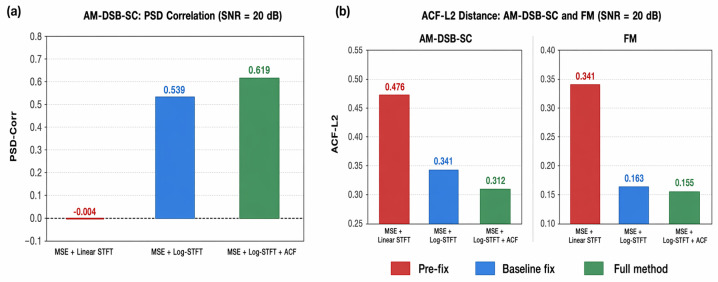
Log-STFT preprocessing resolves AM generation failure. Linear STFT produces anti-correlated spectra; logarithmic compression restores spectral fidelity; adding ACF regularization yields further improvement.

These results confirm the gradient imbalance diagnosis: for analog modulations, linear STFT creates conditions under which the spectral loss overwhelms temporal supervision, and log compression provides an effective correction. Figure 6 illustrates the effect for AM: linear STFT yields anti-correlated spectra, Log-STFT restores the spectral envelope, and adding ACF regularization further sharpens the temporal structure. The improvement observed here is consistent with patterns documented in preliminary experiments, in which linear STFT configurations consistently exhibited elevated ACF-L2 for amplitude-modulated signals relative to their Log-STFT counterparts.

### 5.4. ε-Prediction vs. $x_{0}$-Prediction

Twenty expert models (10 modulations × 2 parameterizations) were trained to compare ε-prediction and $x_{0}$-prediction. Both variants use Log-STFT preprocessing. Table 5 presents the ACF-L2 comparison at SNR =20 dB.

The results exhibit a clear modulation-dependent pattern. For the two analog modulations, $x_{0}$-prediction produces large improvements: AM ACF-L2 reduces from 0.594 to 0.024 (96% improvement), and FM improves from 0.203 to 0.045 (78% improvement). OOK, the other amplitude-varying modulation, improves by 35%. These gains are accompanied by PSD correlations of 0.990–1.000, indicating that $x_{0}$-prediction improves temporal structure without sacrificing spectral fidelity.

**Figure 7:**
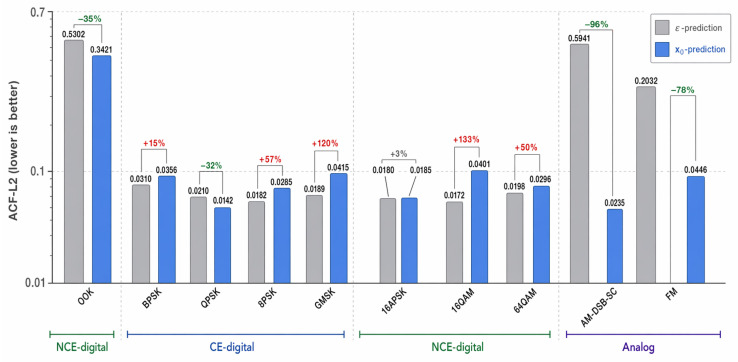
ACF-L2 comparison: ε-prediction vs. $x_{0}$-prediction at SNR = 20 dB across ten modulation types. Analog and amplitude-varying signals (AM, FM, OOK) improve 35–96%; most digital modulations show modest trade-offs within the acceptable range.

For the seven digital modulations, the picture is more nuanced. BPSK, 8PSK, 16QAM, 64QAM, and GMSK show increased ACF-L2 under $x_{0}$-prediction, with increases ranging from 16% to 135%. QPSK shows a notable improvement, while 16APSK is essentially unchanged. Importantly, all $x_{0}$-prediction ACF-L2 values remain within an acceptable range (0.014–0.042), and PSD correlations remain above 0.86 for all modulations. Figure 7 summarizes the comparison: analog and amplitude-varying modulations (AM, FM, OOK) move toward lower ACF-L2 under $x_{0}$-prediction, while most digital modulations show small increases that remain within the calibrated acceptable region.

This pattern is consistent with the SNR-dependent weight analysis (Section 4.4.2): $x_{0}$-prediction’s uniform weighting is essential for analog and amplitude-varying signals but represents a trade-off for digital modulations; ε-prediction’s emphasis on low-noise fine-structure recovery provides a modest advantage in that regime.

**Figure 8:**
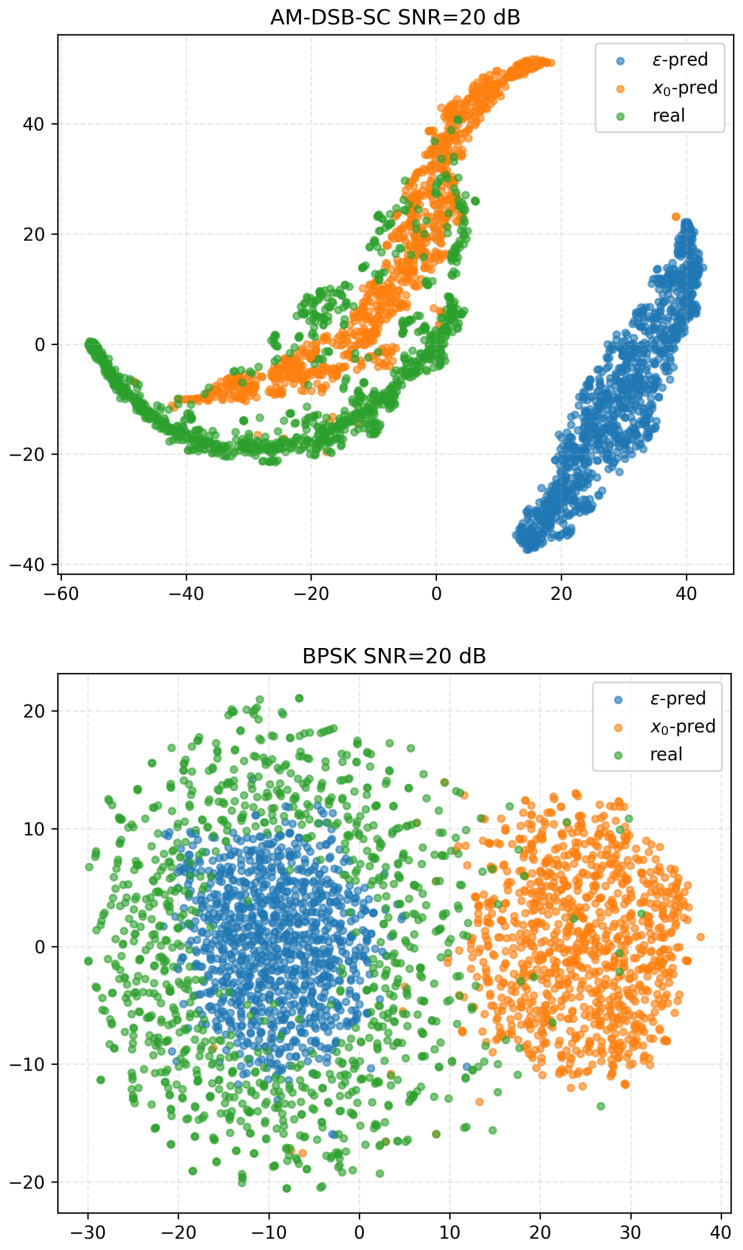
t-SNE embeddings of per-sample ACF vectors (SNR = 20 dB). Top: AM-DSB-SC; bottom: BPSK. For AM-DSB-SC, $x_{0}$-prediction (orange) overlaps with real data (green), while ε-prediction (blue) is separated. For BPSK, ε-prediction is closer to real data, illustrating the modulation-dependent trade-off.

Figure 8 provides feature-space evidence for this trade-off. Using per-sample autocorrelation vectors as input to t-SNE, the $x_{0}$-prediction-generated distribution for AM-DSB-SC overlaps substantially with the real-data distribution, while the ε-prediction-generated distribution forms a separate cluster. For BPSK, the relationship is reversed: ε-prediction lies closer to the real distribution, while $x_{0}$-prediction forms a distinct cluster, consistent with the slightly higher ACF-L2 reported in Table 5. These embeddings confirm that the metric differences reflect genuine distributional shifts rather than statistical fluctuations in the ACF-L2 estimate.

### 5.5. ACF Regularization Effects

**Figure 9:**
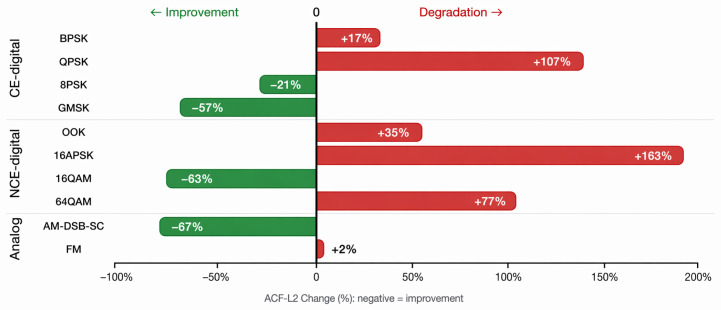
Effect of ACF regularization ($\lambda_{\mathrm{acf}}=0.05$) under $x_{0}$-prediction. Four modulations improve, five degrade, and FM is essentially unchanged—no statistically significant directional effect, indicating that a uniform ACF weight is suboptimal.

Table 6 and Figure 9 report the effect of ACF regularization under $x_{0}$-prediction across all ten modulations. The results reveal modulation-dependent effects with no consistent pattern: AM benefits substantially (ACF-L2 reduced from 0.024 to 0.008), while 16APSK degrades markedly (0.019 to 0.050). Across the ten modulations, four improve, five degrade, and FM remains essentially unchanged (0.045 to 0.046) with ACF regularization, yielding no statistically significant directional effect. This heterogeneity indicates that a uniform ACF weight ($\lambda_{\mathrm{acf}}=0.05$) is suboptimal for multi-modulation training, and per-family or adaptive ACF weighting strategies may be necessary. We report these findings as an open challenge identified by the evaluation framework.

## 6. Discussion

### 6.1. Framework as Diagnostic Tool

The evaluation framework’s primary contribution is its capacity to systematically reveal generation quality issues invisible to conventional metrics. The PSD correlation metric remained above 0.86 for all modulations under both parameterizations, suggesting comparable spectral fidelity; the ACF-L2 metric, operating in the orthogonal temporal dimension, revealed substantial differences entirely obscured by spectral measures alone. Unlike existing approaches that aggregate quality into a single score, such as the Inception Score and FID in image generation [16] or downstream classification accuracy in RF-Diffusion [5], our framework provides per-dimension calibrated metrics that pinpoint specific failure modes. The Log-STFT and $x_{0}$-prediction improvements documented here reduce the ACF-L2 gap to the real-real baseline rather than merely shifting error between metrics.

### 6.2. Implications for Practice

Practitioners generating analog or amplitude-varying signals (e.g., AM, FM, and OOK) should prefer $x_{0}$-prediction to avoid the temporal structure degradation that ε-prediction induces. For most digital communication signals (BPSK, 8PSK, GMSK, 16QAM, and 64QAM), ε-prediction with Log-STFT preprocessing remains the better choice in terms of ACF-L2 and PSD correlation, although QPSK benefits from $x_{0}$-prediction (see Table 5).

### 6.3. Generality and Applicability

The framework is model-agnostic: it consumes generated and authentic complex baseband I/Q samples and requires no access to the generator’s architecture, loss function, or training procedure. Consequently, it applies directly to GANs, VAEs, diffusion models, autoregressive transformer models, and any other generative approach that outputs I/Q waveforms (Figure 1). For models that natively produce discrete tokens or latent representations, a decoding step is required to recover I/Q samples, after which the framework is applied unchanged.

We deliberately do not adopt FID, IS, or LPIPS as primary metrics because they rely on ImageNet-pretrained visual features or human-perceptual distances that have no physical interpretation for RF signals. Training an RF-specific feature extractor would create a circular dependency and compress multidimensional quality into an uninterpretable scalar. Instead, the proposed metrics operate directly on the I/Q waveform and preserve physical interpretability.

The same holds for datasets: the metrics depend only on the I/Q time series and on modulation/SNR labels for stratification, so they can be recomputed on any RF dataset (synthetic or recorded) without modification. Multi-antenna or wideband signals would require extending PSD and ACF computations to spatial or additional frequency dimensions, but the four design principles remain valid. Although the present experiments use communication-style modulation data, the same I/Q-waveform primitives underlie radar, spectrum sensing, and RF fingerprinting signals. The framework therefore transfers directly to those sensor domains provided appropriate stratification labels are available (e.g., waveform type, target class, or device ID); this study establishes the evaluation methodology and calibration protocol, while domain-specific empirical validation remains for future work.

### 6.4. Limitations

Our study has several limitations. First, the experiments focus on RML2018’s ten-modulation subset; extension to the full 24-modulation dataset and to over-the-air captured signals would further validate generalizability. The metrics are dataset-agnostic in principle—they require only I/Q samples and stratification labels—but additional empirical validation on datasets such as HisarMod2019.1 and TorchSig, and on real measured RF recordings, remains future work. To mitigate this within the available data, we report within-RML2018 robustness checks: the calibration subsets are re-sampled, and the framework is evaluated on held-out splits, confirming that normalized scores are stable to the choice of calibration data (see Section 5.2). The single-modulation training protocol cleanly isolates modulation-specific effects, yet it does not reflect practical multi-modulation training, in which one conditional model generates diverse modulation types.

Second, the ACF regularization component shows modulation-dependent effects that we do not fully resolve; adaptive or per-family ACF weighting may be necessary. Third, cyclostationary analysis through the spectral correlation function (SCF) remains unexplored; the SCF can detect periodic structures invisible to stationary analysis [23] and may add diagnostic resolution for modulation-specific artifacts. Fourth, the *v*-prediction parameterization [8] offers a principled alternative that avoids the ε-prediction gradient suppression without sacrificing low-noise structure recovery, and evaluating it for RF signals is an important future direction. Fifth, training 20 expert models is computationally expensive and limits the statistical power of our comparisons; larger-scale training runs could quantify parameterization effects more precisely. Sixth, although the framework is motivated by and applicable to sensor systems in principle, all empirical experiments use RML2018 communication modulation data. Direct validation on radar, spectrum-sensing, or RF-fingerprinting datasets would strengthen the practical claims for sensor applications and is left for future work.

## 7. Conclusions

We presented a multi-dimensional, distribution-level evaluation framework for RF signal generation with diffusion models, employing ten metrics across six layers calibrated against real-real baselines. Application across ten modulation types and two training parameterizations revealed two previously unreported phenomena: a gradient imbalance caused by linear STFT preprocessing that logarithmic compression resolves and a systematic gradient suppression in ε-prediction that $x_{0}$-prediction addresses. The framework’s theoretical foundations—the periodogram estimator’s $\chi_{2}^{2}$-limited per-sample correlation ceiling and the SNR-dependent implicit loss weighting analysis—provide rigorous interpretability for all metrics.

The framework and calibration methodology extend to other generative architectures and RF signal types. We will release the calibration data and evaluation code upon publication. Key directions for future work include evaluating the *v*-prediction parameterization [8], extending the framework with cyclostationary analysis [24], and validating on multi-modulation joint training and over-the-air signals beyond the RML2018 dataset.

## Figures and Tables

**Table 1:** Multi-dimensional evaluation framework. All metrics are distribution-level, computed per-(modulation, SNR) cell, and normalized by real-real baselines.

Layer	Metric	Computation	Diagnostic Purpose
L0	MSE	Mean paired squared L2 distance	Failure detection
L0	CosSim	Mean paired cosine similarity	Waveform bias check
L1	PSD Correlation	Pearson *r* of avg. Welch PSDs	Spectral shape fidelity
L1	Spectral Convergence	L2 of avg. log-STFT matrices	Fine time-frequency structure
L2	ACF-L2 (mag.)	L2 of avg. ACF magnitude vectors	Temporal structure (core)
L2	ACF-L2 by lag	Decomposed: near/mid/far τ	Multi-scale diagnostics
L3a	EVM Ratio	RMS spread vs. ideal constellation	Constellation quality (digital)
L3b	Envelope W1	1D Wasserstein of envelope dist.	AM fidelity
L3b	Instantaneous Frequency (IF) W_1_	1D Wasserstein of inst. freq. dist.	FM fidelity
L4	Kurtosis (I/Q)	Excess kurtosis of pooled I and Q	Modulation fingerprint

**Table 2:** Metrics excluded from the framework with justifications.

Excluded Metric	Reason for Exclusion
Per-sample PSD-Corr	Theoretical ceiling of 0.5 for digital modulations (see Section 4.4.1) precludes cross-modulation comparison.
Phase ACF	Random symbol phases dominate the phase ACF; the real-real baseline already spans nearly the full dynamic range, leaving little discriminative power for generation-quality differences.
FID/IS/Precision-Recall/LPIPS	Depend on ImageNet-pretrained features or human-visual distances with no RF physical interpretation. Training an RF-specific extractor would create a circular dependency and compress quality into an uninterpretable scalar.
SSIM	Designed for human visual perception; luminance-contrast-structure decomposition has no physical interpretation for I/Q samples.
IQ KL Divergence	Replaced by cumulants, which provide equivalent distributional verification with stronger diagnostic interpretability (Section 4.2).
Constellation EMD	2D Wasserstein distance of I/Q point clouds is redundant with EVM ratio for constellation quality assessment.
Symbol Error Rate	Requires ground-truth bit sequences not available in the RML2018 dataset.

**Table 3:** Real-real calibration baselines at SNR =20 dB (N=2048 per half). Based on variance observed across calibration subsets, we estimate standard errors of approximately ±15% of reported ACF-L2 values for digital modulations and ±25% for analog. PSD-Corr SE $<10^{-4}$. EVM is N/A for modulations without fixed constellation points. SC: Spectral Convergence; Env-W_1_: Envelope Wasserstein distance; IF-W_1_: Instantaneous Frequency Wasserstein distance; Kurtosis (I): I-channel excess kurtosis.

Modulation	PSD-Corr	SC	ACF-L2	EVM	Env-W_1_	IF-W_1_	Kurtosis (I)
OOK	0.999	0.011	0.0025	N/A	0.004	0.002	+0.15
BPSK	1.000	0.009	0.0018	0.99	0.002	0.001	−0.71
QPSK	1.000	0.008	0.0013	1.00	0.003	0.001	−1.11
8PSK	1.000	0.009	0.0010	1.00	0.003	0.001	−1.11
16APSK	1.000	0.009	0.0013	1.00	0.003	0.001	−0.90
16QAM	1.000	0.008	0.0012	1.00	0.002	0.001	−0.76
64QAM	1.000	0.008	0.0013	0.99	0.004	0.001	−0.69
AM	1.000	0.011	0.0001	N/A	0.004	0.001	−0.65
FM	1.000	0.009	0.0087	N/A	0.000	0.000	−1.50
GMSK	1.000	0.009	0.0014	N/A	0.001	0.001	−1.45

**Table 4:** Log-STFT vs. linear STFT for analog modulations (SNR =20 dB, ε-prediction). $L_{\mathrm{pred}}$ denotes the time-domain prediction loss (MSE against the prediction target), not the L0 evaluation metric.

Configuration	AM PSD-Corr	AM ACF-L2	FM ACF-L2
$L_{\mathrm{pred}}$ + linear STFT	−0.004	0.476	0.341
$L_{\mathrm{pred}}$ + Log-STFT	0.539	0.341	0.163
$L_{\mathrm{pred}}$ + Log-STFT + ACF	0.619	0.312	0.155

**Table 5:** ACF-L2 comparison: ε-prediction vs. $x_{0}$-prediction (SNR =20 dB). Raw ACF-L2 values. Estimated SE ≈±15% for digital, ±25% for analog based on calibration subset variance. Improvement computed as $(x_{0}-\varepsilon )/\varepsilon \times 100\%$; negative indicates better $x_{0}$. Bold values indicate the modulation with the largest improvement (AM-DSB-SC).

Modulation	ε-ACF	$x_{0}$-ACF	Change	$x_{0}$-PSD
OOK	0.530	0.342	−35%	0.990
BPSK	0.031	0.036	+16%	0.881
QPSK	0.021	0.014	−33%	0.988
8PSK	0.018	0.029	+61%	0.926
16APSK	0.018	0.019	+6%	0.971
16QAM	0.017	0.040	+135%	0.862
64QAM	0.020	0.030	+50%	0.925
**AM**	**0.594**	**0.024**	**−96%**	**1.000**
FM	0.203	0.045	−78%	1.000
GMSK	0.019	0.042	+121%	0.885

**Table 6:** Effect of ACF regularization under $x_{0}$-prediction (SNR =20 dB). Baseline: no ACF loss. With ACF: $\lambda_{\mathrm{acf}}=0.05$. Standard errors estimated from calibration subset variance are approximately ±15% of reported ACF-L2 for digital modulations and ±25% for analog. Bold values indicate the modulation with the largest improvement (AM-DSB-SC).

Modulation	No ACF	With ACF	Change
OOK	0.342	0.460	+35%
BPSK	0.036	0.042	+17%
QPSK	0.014	0.029	+107%
8PSK	0.029	0.023	−21%
16APSK	0.019	0.050	+163%
16QAM	0.040	0.015	−63%
64QAM	0.030	0.053	+77%
**AM**	**0.024**	**0.008**	**−67%**
FM	0.045	0.046	+2%
GMSK	0.042	0.018	−57%

## Data Availability

The RML2018 dataset is publicly available. Extracted data subsets, evaluation code, and real-real calibration results will be released upon publication.

## List of Acronyms

ACF	Autocorrelation Function
AdaLN	Adaptive Layer Normalization
AMC	Automatic Modulation Classification
CosSim	Cosine Similarity
DDIM	Denoising Diffusion Implicit Model
DDPM	Denoising Diffusion Probabilistic Models
DiT	Diffusion Transformer
EVM	Error Vector Magnitude
FAD	Fréchet Audio Distance
FID	Fréchet Inception Distance
IF	Instantaneous Frequency
I/Q	In-phase/Quadrature
IS	Inception Score
LPIPS	Learned Perceptual Image Patch Similarity
MCD	Mel Cepstral Distortion
MSE	Mean Squared Error
PSD	Power Spectral Density
RF	Radio Frequency
SC	Spectral Convergence
SCF	Spectral Correlation Function
SER	Symbol Error Rate
SI-SDR	Scale-Invariant Signal-to-Distortion Ratio
SNR	Signal-to-Noise Ratio
SSIM	Structural Similarity Index Measure
STFT	Short-Time Fourier Transform
